# Salvage treatment patterns in multiple myeloma patients progressing after lenalidomide maintenance: A real‐life study from European Myeloma Network (EMN) Italy

**DOI:** 10.1111/bjh.70751

**Published:** 2026-08-18

**Authors:** Gregorio Barilà, Angela Grassi, Valeria Ruocco, Carmine Liberatore, Edoardo Olivari, Martina Tinelli, Francesca Fazio, Sonia Morè, Emanuele Favero, Claudio Salvatore Cartia, Nicola Sgherza, Concetta Conticello, Roberto Mina, Martina Gherardini, Giuseppe Mele, Massimo Gentile, Anna Furlan, Massimiliano Arangio Febbo, Maria Livia Del Giudice, Ombretta Annibali, Bernardo Rossini, Edoardo Scomazzon, Marco Talarico, Enrica Antonia Martino, Claudio De Magistris, Francesca Fioritoni, Anna Maria Cafro, Laura Paris, Sara Pezzatti, Roberta Della Pepa, Paola Boggione, Giuseppe Pietrantuono, Valeria Tomarchio, Francesco Vassallo, Maria Luisa Pioltelli, Francesco Pisani, Antonia Cagnetta, Lorenzo De Paoli, Gabriele Buda, Elisabetta Antonioli, Monica Galli, Alberto Tosetto, Pellegrino Musto, Elena Zamagni, Francesca Patriarca, Silvia Mangiacavalli, Renato Zambello, Massimo Offidani, Maria Teresa Petrucci

**Affiliations:** ^1^ Hematology Unit San Bortolo Hospital Vicenza Italy; ^2^ Clinical Research Unit Veneto Institute of Oncology, IOV‐IRCCS Padua Italy; ^3^ Department of Medicine (DIMED), Hematology and Clinical Immunology Section Padua University School of Medicine Padua Italy; ^4^ Department of Medicine and Sciences of Aging “G d'Annunzio” University Chieti Italy; ^5^ Hematology Unit Santo Spirito Hospital Pescara Italy; ^6^ Hematology and Bone Marrow Transplant Unit ASST Papa Giovanni XXIII Bergamo Italy; ^7^ Department of Medicine, Section of Hematology University of Verona Verona Italy; ^8^ Division of Hematology, Department of Translational and Precision Medicine Azienda Ospedaliera Universitaria Policlinico Umberto I, Sapienza University of Rome Rome Italy; ^9^ Hematology Unit AOU delle Marche Ancona Italy; ^10^ Hematology and Transplant Center Unit, Udine University Hospital, DMED University of Udine Udine Italy; ^11^ Division of Hematology Fondazione IRCCS Policlinico San Matteo Pavia Italy; ^12^ Hematology and Stem Cell Transplantation Unit AOU Consorziale Policlinico Bari Italy; ^13^ Division of Hematology Azienda Policlinico‐S.Marco, University of Catania Catania Italy; ^14^ Division of Hematology Azienda Ospedaliero‐Universitaria Città della Salute e della Scienza di Torino Turin Italy; ^15^ Haematology and Stem Cell Transplant Unit Ospedale Antonio Perrino Brindisi Italy; ^16^ Hematology Unit, Department of Onco‐Hematology A.O. of Cosenza Cosenza Italy; ^17^ Department of Pharmacy, Health and Nutritional Science University of Calabria Rende Italy; ^18^ Hematology Unit Ospedale Santa Maria di Ca' Foncello Treviso Italy; ^19^ Onco Hematology Unit Veneto Institute of Oncology, IOV‐ IRCCS Padua Italy; ^20^ Department of Clinical and Experimental Medicine, Hematology University of Pisa Pisa Italy; ^21^ Hematology and Stem Cell Transplantation Unit Campus Bio‐Medico University Rome Italy; ^22^ Haematology and Transplant Unit IRCCS ‐ Istituto Tumori “Giovanni Paolo II” Bari Italy; ^23^ IRCCS Azienda Ospedaliero‐Universitaria di Bologna Istituto di Ematologia Seràgnoli Bologna Italy; ^24^ Dipartimento di Scienze Mediche e Chirurgiche Università di Bologna Bologna Italy; ^25^ Hematology Unit Fondazione IRCCS Ca' Granda Ospedale Maggiore Policlinico Milan Italy; ^26^ Hematology Unit ASST GOM Niguarda Milan Italy; ^27^ Fondazione IRCCS San Gerardo Monza Italy; ^28^ Department of Clinical Medicine and Surgery University Federico II Naples Italy; ^29^ Division of Hematology, Department of Translational Medicine Università del Piemonte Orientale Novara Italy; ^30^ IRCCS CROB Referral Cancer Center of Basilicata Rionero in Vulture Italy; ^31^ Division of Hematology Ospedale Santa Croce e Carle Cuneo Italy; ^32^ Hematology Unit IRCCS Regina Elena National Cancer Institute Rome Italy; ^33^ IRCCS AOU San Martino‐IST Genoa Italy; ^34^ Hematology Unit Vercelli Italy; ^35^ Hematology Unit Careggi Hospital Florence Italy; ^36^ Department of Precision and Regenerative Medicine and Ionian Area “Aldo Moro” University School of Medicine Bari Italy

**Keywords:** anti‐CD38 based combinations, lenalidomide maintenance, lenalidomide refractory, multiple myeloma

## Abstract

The extensive use of lenalidomide (Len) in the first‐line treatment of multiple myeloma (MM) has resulted in an increased frequency of patients who have been exposed or are refractory to Len (Len‐R) at first relapse. However, the outcome of Len‐R patients treated at first relapse after autologous haematopoietic stem cell transplantation (AHSCT) remains unclear. The aim of this real‐life study is to evaluate the effectiveness of the regimens licensed in Italy and used in a cohort of patients who relapsed during Len maintenance after AHSCT. We collected 306 patients with these features. Considering the different regimens used, most patients received isatuximab–carfilzomib–dexamethasone (Isa‐KD) (45.4%) followed by daratumumab–pomalidomide–dexamethasone (D‐PD) (19.3%) and daratumumab–bortezomib–dexamethasone (D‐VD) (12.4%). Overall, a reduced progression‐free survival (PFS) and overall survival (OS) were observed in patients with high‐risk disease features at relapse, including high lactate dehydrogenase (LDH) (*p* = 0.0003 and *p* < 0.0001), International Staging System (ISS) ≥2 (*p* < 0.0001 and *p* < 0.0001) and early relapse (<12 months from maintenance initiation) (*p* = 0.0017 and *p* = 0.0009). Considering the type of treatment, anti‐cluster of differentiation 38 (CD38) monoclonal antibodies (anti‐CD38 MoAb)‐based regimen displayed improved survival compared to the other treatment, with Isa‐KD reporting the longest PFS (22.8 months). Our data in a homogenous cohort of Len‐R patients treated at first relapse demonstrated that the anti‐CD38 MoAb‐based combinations, particularly Isa‐KD, represented the standard of care before the approval of anti‐B cell maturation antigen (BCMA) immunotherapy.

## INTRODUCTION

The extensive use of lenalidomide (Len) in the first‐line treatment of multiple myeloma (MM), either as post‐autologous haematopoietic stem cell transplantation (AHSCT) maintenance or in combination with dexamethasone (RD) for transplant‐ineligible patients, has resulted in an increased frequency of patients who have been exposed or, most importantly, are refractory to Len (Len‐R) at first relapse.^1^, ^2^

Over the years, several combinations have been subsequently exploited for the treatment of Len‐R. In this setting, the advent of anti‐CD38 monoclonal antibodies (anti‐CD38 MoAb) in combination with carfilzomib or pomalidomide has significantly improved the outcome of Len‐R patients across all trials. The newest combinations of daratumumab–carfilzomib–dexamethasone (D‐KD) and isatuximab–carfilzomib–dexamethasone (Isa‐KD) became the new standard of care for these patients, with the unprecedented progression‐free survival (PFS) of 28.1 months reported in the CANDOR trial,^3^, ^4^ and the significant benefit of the triplet regimen in the IKEMA trial as well in all the patients' subgroups.^5^, ^6^, ^7^, ^8^, ^9^, ^10^ However, only the Isa‐KD regimen has been licensed in Italy. More recently, the combination of daratumumab–pomalidomide–dexamethasone (D‐PD) became available for the treatment of MM patients at first relapse given the results of the APOLLO trial, with a median PFS of 9.9 months in Len‐R patients,^11^, ^12^ and the more impressive PFS of 21.8 months reported in the phase‐2 MM‐014 trial.^13^, ^14^

Notably, in all the randomized clinical trials that evaluated the regimens used in MM patients relapsing after Len, the proportion of Len‐R cases was highly variable and never encompassed the whole cohort. Moreover, most patients were treated at second relapse without specifying whether they received Len in combination or as maintenance after AHSCT. Consequently, the outcome of Len‐R patients after AHSCT and maintenance remains unclear.

The aim of this real‐life study is to evaluate the effectiveness of past and currently approved regimens in Italy and used in a homogeneous cohort of patients who relapsed during Len maintenance after AHSCT.

## METHODS

### Study design and patients

The PALLADIO study (VI‐MM‐02) is a retrospective and prospective cohort study involving 31 Italian centres and enrolling patients with relapsed and refractory MM who progressed after Len maintenance and received any treatment starting from 2 November 2017.

Per protocol design, all patients received at least one AHSCT (single or tandem AHSCT according to local clinical practice), Len maintenance until progression and treatment at first relapse.

Demographic, clinical and biological characteristics were collected, including International Staging System (ISS), cytogenetic analysis, isotype and symptoms at the time of diagnosis and first relapse. Extra‐medullary disease (EMD) was defined as bone‐independent soft‐tissue plasmocytoma.

The response rate was determined according to the International Myeloma Working Group criteria. Grading of adverse events (AEs) was evaluated by each clinician through the Common Terminology Criteria for Adverse Events (CTCAE) Version 5.0.

This study was approved by the Local Ethical Committee of the South‐West Veneto Region (CET‐ASOV) and conducted in accordance with the Declaration of Helsinki. All patients provided written informed consent prior their inclusion.

### Statistical analysis

Descriptive statistics were reported as median (interquartile range, Q1–Q3 and range, min–max) for continuous variables and frequency (percentage) for categorical variables. For descriptive purposes, relapse time from maintenance initiation was discretized into three levels (<12, 12–24, >24 months). The patient's overall survival (OS) was calculated from the date of treatment initiation after relapse to death by any cause or to the last known follow‐up visit for censored patients. PFS was defined as the time from treatment initiation after relapse to tumour progression or death by any cause, with censoring of patients lost to follow‐up. Survival curves were estimated using the Kaplan–Meier method and significant differences between groups were tested using the two‐tailed log‐rank test.

For a more straightforward interpretation of hazard ratios (HRs), non‐binary variables were dichotomized as follows: age at treatment initiation after relapse (>65 vs. ≤65 years), relapse time from maintenance initiation (>24 vs. ≤24 months), cytogenetic status at relapse (HR vs. SR), number of cytogenetic abnormalities (≥2 vs. <2), lactate dehydrogenase (LDH) at relapse (high vs. low), ISS at relapse (II–III vs. I), estimated glomerular filtration rate (eGFR, ≥60 vs. <60 mL/min). Schoenfeld residual testing was applied to assess the assumption of proportional hazards. A univariable Cox proportional hazards regression analysis was employed to evaluate the prognostic relevance of each variable. A multivariable Cox proportional hazards regression analysis was conducted, considering as covariates the variables with statistically significant impact (*p*‐value <0.05) in the univariate analysis and performing a backward elimination to retain only those with independent prognostic value. Results were presented as HRs and 95% confidence intervals. Statistical analysis was conducted in R version 4.5.1.

## RESULTS

### Demographics and characteristics

We analysed 306 MM patients who started any second line treatment after AHSCT and Len maintenance until progression between 2 November 2017 and 14 March 2025. The study cohort's demographics, clinical and biological characteristics at diagnosis and relapse are reported in Table 1. The median age was 63 years (range 33–77), and 131/305 cases (43%) were >65 years old. Considering first‐line treatment, almost all patients received at least triplet‐based induction, mostly bortezomib–thalidomide–dexamethasone (VTD, 82%), with only 18/306 (5.9%) treated with an anti‐cluster of differentiation 38 (CD38) MoAb‐based quadruplet. In 196/306 (64.1%) patients a single AHSCT was performed while the remaining 110/306 (35.9%) received tandem AHSCT. As per protocol design, all patients received Len maintenance until progression, with a median treatment duration of 19.5 months.

**TABLE 1:** Demographics, biological and clinical characteristics of the study cohort.

Characteristic	Cohort (*n* = 306)
Gender Male—no./total no. (%)	160/306 (52.3%)
Age at treatment initiation after relapse, years	
Median [Q1; Q3] [min; max]	63 [57;68][33;77]
Age at treatment initiation after relapse >65 years—no./total no. (%)	131/305 (43.0%)
Induction therapy	
VTD—no./total no. (%)	251/306 (82.0%)
D‐VTD—no./total no. (%)	15/306 (4.9%)
VCD—no./total no. (%)	10/306 (3.3%)
VRD—no./total no. (%)	8/306 (2.6%)
KRD—no./total no. (%)	7/306 (2.3%)
KCD—no./total no. (%)	2/306 (0.7%)
D‐VRD—no./total no. (%)	2/306 (0.7%)
D‐VCD—no./total no. (%)	1/306 (0.3%)
Others^a^—no./total no. (%)	10/306 (3.2%)
Previous anti‐CD38‐based therapy (D‐VTD; D‐VRD; D‐VCD)—no./total no. (%)	18/306 (5.9%)
Number of autoHSCTs	
1—no./total no. (%)	196/306 (64.1%)
2—no./total no. (%)	110/306 (35.9%)
Maintenance duration, months—Median [Q1;Q3] [min; max]	19.5[11.9;33.7][0.9;113.8]
Relapse time from maintenance initiation, months	
<12—no./total no. (%)	78/306 (25.5%)
12–24—no./total no. (%)	100/306 (32.7%)
>24—no./total no. (%)	128/306 (41.8%)
Cytogenetic status at relapse	
HR [del(17p),t(4;14),t(14;16),+1q]—no./total no. (%)	90/143 (62.9%)
HR [del(17p),t(4;14),t(14;16)]—no./total no. (%)	50/143 (35.0%)
SR—no./total no. (%)	93/143 (65.0%)
Isolated +1q—no./total no. (%)	40/90 (44.4%)
Number of cytogenetic abnormalities	
0—no./total no. (%)	53/143 (37.1%)
1—no./total no. (%)	57/143 (39.9%)
≥2—no./total no. (%)	33/143 (23.1%)
ISS (at relapse)—no./total no. (%)	
I	121/174 (69.5%)
II–III	53/174 (30.5%)
LDH high (at relapse)—no./total no. (%)	53/279 (19.0%)
eGFR ≥ 60—no./total no. (%)	236/290 (81.4%)
Type of relapse	
Biochemical—no./total no. (%)	120/306 (39.2%)
Symptomatic—no./total no. (%)	186/306 (60.8%)
Extra‐medullary disease—no./total no. (%)	17/300 (5.7%)

*Note*: Percentages might not sum to 100 because of rounding. Denominator might not be equal to 306 due to data missing.

Abbreviations: autoHSCT, autologous haematopoietic stem cell transplantation; eGFR, estimated glomerular filtration rate; HR, high‐risk; ISS, international staging system; SR, standard risk.

^a^ Including VD, VTD‐PACE, TD, RD, DCEP, CTD.

With respect to high‐risk disease features at relapse, high LDH levels and ISS ≥ II were present in 53/279 (19.0%) and 53/174 (30.5%) of patients, respectively, while high‐risk (HR) cytogenetic abnormalities (including t(4;14), t(14,16), del17p and +1q) were present in 90/143 (62.9%) patients, of whom 40/90 (44.4%) had isolated +1q abnormalities (either gain or amp1q). Moreover, in 23.1% of cases (33/143) at least 2 HR cytogenetic abnormalities were identified.

Most patients were treated due to symptomatic relapse 186/306 (60.8%), with 17/300 (5.7%) displaying EMD, while the remaining 120 cases (39.2%) started a second line treatment due to biochemical relapse.

### Treatments received by the study cohort

Considering the different regimens used, almost half of patients received Isa‐KD 139/306 (45.4%) followed by D‐PD 59/306 (19.3%) and daratumumab–bortezomib–dexamethasone (D‐VD) 38/306 (12.4%). Only few patients were treated with pomalidomide–bortezomib–dexamethasone (P‐VD) or KD (24/306, 7.8% and 19/306, 6.2% respectively) whereas 20/306 (6.5%) received Len‐based triplets, mostly daratumumab–lenalidomide–dexamethasone (D‐RD) (Table 2).

**TABLE 2:** Therapy of patients progressing after lenalidomide maintenance.

Characteristic	Cohort (*n* = 306)
Type of therapy	
Isa‐KD—no./total no. (%)	139/306 (45.4%)
D‐PD—no./total no. (%)	59/306 (19.3%)
D‐VD—no./total no. (%)	38/306 (12.4%)
P‐VD—no./total no. (%)	24/306 (7.8%)
KD—no./total no. (%)	19/306 (6.2%)
D‐RD—no./total no. (%)	14/306 (4.6%)
K‐RD—no./total no. (%)	6/306 (2.0%)
Other—no./total no. (%)	7/306 (2.3%)
Anti‐CD38‐based combinations (Isa‐KD; D‐VD; D‐PD; D‐RD)—no./total no. (%)	250/306 (81.7%)
PI+anti‐CD38 (Isa‐KD; D‐VD)—no./total no. (%)	177/306 (57.8%)
IMIDs+anti‐CD38 (D‐PD; D‐RD)—no./total no. (%)	73/306 (23.9%)
Len‐based triplets (D‐RD; K‐RD; Ixa‐RD)—no./total no. (%)	20/306 (6.5%)
Number of cycles administered—Median [Q1;Q3] [min; max]	9[5;18][1;78]
ORR—no./total no. (%)	246/298 (82.6%)
Complete response (sCR; CR)—no./total no. (%)	81/298 (27.2%)
Transplantation in relapse	
AutoHSCT—no./total no. (%)	15/305 (4.9%)
AlloHSCT—no./total no. (%)	4/305 (1.3%)
None—no./total no. (%)	286/305 (93.8%)

*Note*: Percentages might not sum to 100 because of rounding. Denominator might not be equal to 306 due to data missing.

Abbreviations: AlloHSCT, allogenic haematopoietic stem cell transplantation; AutoHSCT, autologous haematopoietic stem cell transplantation; IMIDs, immunomodulatory drugs; Len, Lenalidomide; ORR, overall response rate; PI, proteasome inhibitor.

More than 80% of cases were treated with an anti‐CD38 MoAb‐based combination, mostly proteasome inhibitor (PI)+anti‐CD38 177/306 (57.8%) while 73/306 (23.9%) with immunomodulatory drugs (IMIDs)+anti‐CD38 regimens. Figure 1 and Table S1 report the distribution of second line treatments in relation to the year of treatment initiation. It is worth noticing that more than 2/3 of the patients relapsed and started treatment after 2022. According to the Italian approval and reimbursement procedures, it is not surprising that most patients received Isa‐KD and D‐PD (approved in early 2022 and early 2023, respectively) while other combinations were preferred in the former.

**FIGURE 1:**
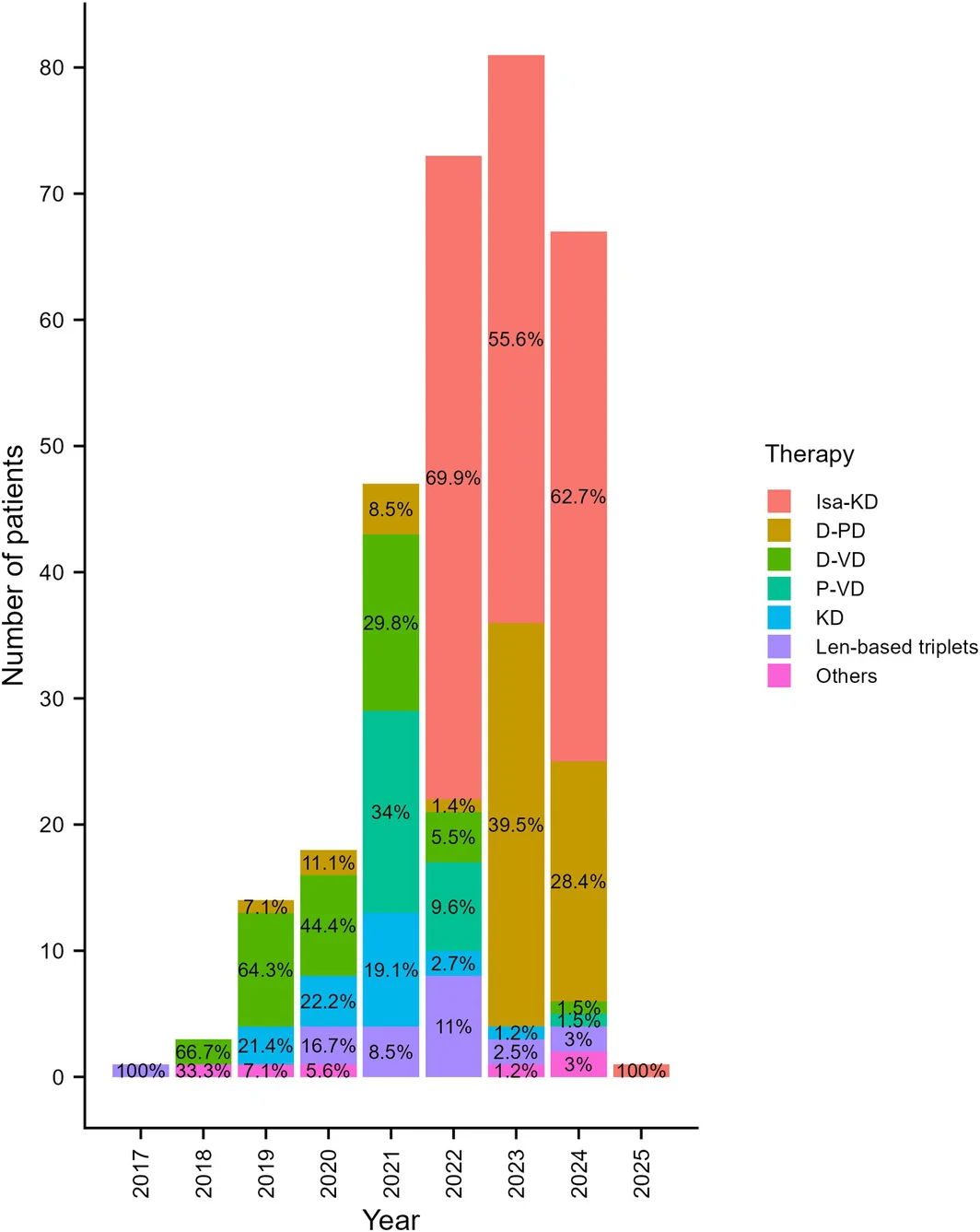
Distribution of different treatments across years in the study cohort. Stacked barplot representing the frequency of the different second line treatments across the years.

With a median number of 9 cycles administered (range 1–78), the overall response rate (ORR) covering all the treatments was 82.6%, with a complete response (CR) plus stringent complete response (sCR) rate of 27.2% (Table 2). Table S2 reported in detail the response rates for each combination. Considering CR+sCR rate, Isa‐KD granted the best outcome (31.3%), followed by D‐PD (26.4%) and D‐VD (26.3%).

Of notice, 19 patients received a haematopoietic stem cell transplantation at relapse, including 15/305 (4.9%) AHSCT and 4/305 (1.3%) allogenic haematopoietic stem cell transplantation (Table 2).

### Survival analyses

With a median follow‐up of 21.2 months, the 2‐years PFS and median PFS were 36.4% and 18.4 months respectively (Figure 2A). With a median follow‐up of 21.6 months, the 2‐years OS and median OS were 72.6% and not reached, respectively (Figure 2B).

**FIGURE 2:**
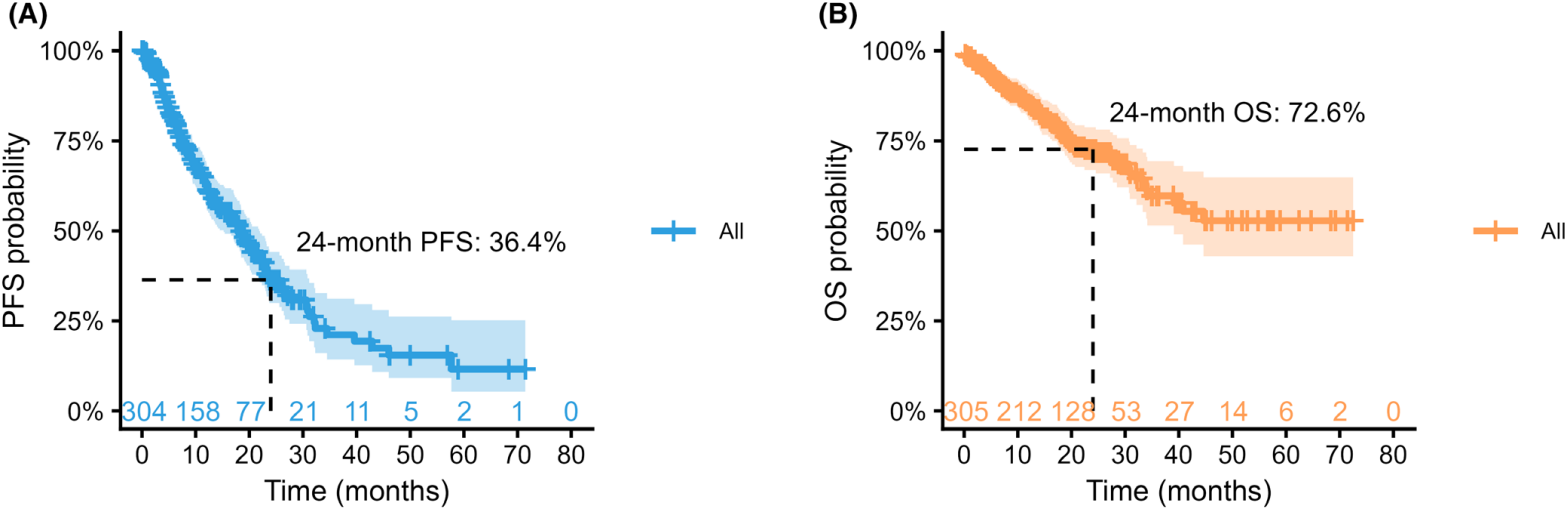
Progression‐free survival and overall survival in the study cohort population. Kaplan–Meier curves showing the Progression‐free survival (PFS) (A) and overall survival (OS) (B) of patients who received a second line treatment after lenalidomide maintenance.

Clinical and biological high‐risk disease proved to confer a dismal outcome in terms of both PFS and OS. In detail, patients with high LDH levels and ISS ≥ II displayed a significantly inferior PFS and OS compared to patients with normal LDH levels and ISS I (median PFS: 7.6 months vs. 20.1 months, *p* = 0.0003 and 8.8 months vs. 23.2 months, *p* < 0.0001, respectively, Figure 3A,C; median OS: 19.5 months vs. not reached, *p* < 0.0001 and 19.5 months vs. not reached, *p* < 0.0001, respectively, Figure 3B,D).

**FIGURE 3:**
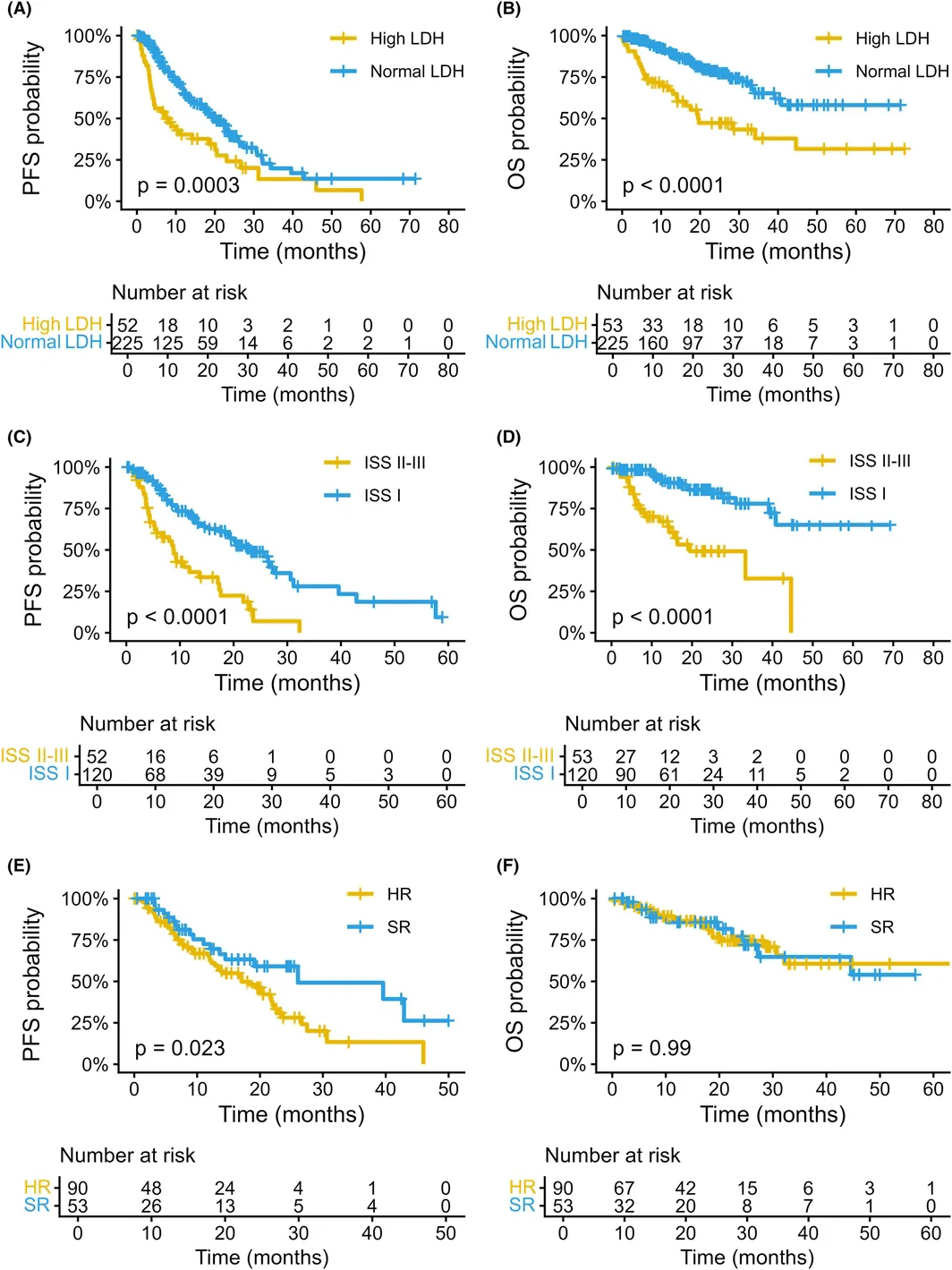
Progression‐free survival and overall survival in different clinical and biological high‐risk subgroups. (A, B) Progression‐free survival (PFS) (A) and overall survival (OS) (B) of patients according to LDH levels. Panels C and D: PFS (C) and OS (D) of patients according to ISS (II–III or I). (E, F) PFS (E) and OS (F) of patients according to cytogenetic risk. Survival curves were estimated using the Kaplan–Meier method and compared by log‐rank test.

The patients harbouring HR cytogenetic abnormalities (including t[4;14], t(14,16), del17p and + 1q) showed reduced PFS compared to SR patients (median PFS: 17.1 months vs. 26 months, *p* = 0.023) while no significant difference in OS was detected (both medians not reached, *p* = 0.99) (Figure 3E,F respectively). Moreover, no significant PFS and OS differences were found in cases harbouring isolated +1q abnormalities compared to SR cases (median PFS: 22.9 months, vs. 26 months, *p* = 0.161; OS: not reached vs. not reached, *p* = 0.31) (Figure S1A,B).

A decreased renal function (eGFR < 60 mL/min) did not significantly impact on PFS (14.2 vs. 19 months, *p* = 0.09) but was significantly associated to reduced OS (both median OS not reached, *p* = 0.0051) (Figure S2A,B). Surprisingly, EMD did not significantly impact on both PFS and OS (median PFS: 10.8 months vs. 18.4 months, *p* = 0.15; both median OS not reached, *p* = 0.96) (Figure S2C,D).

Functional HR confirmed to be a dismal prognostic factor as well. As a matter of fact, patients with early relapse (<12 months of Len maintenance) displayed inferior PFS and OS compared to those who relapsed between 12 and 24 months of maintenance and those with late relapse (>24 months of maintenance) (median PFS: 9.3 months vs. 19.1 months, *p* = 0.0365 and 9.3 months vs. 22.9 months, *p* = 0.0017, respectively; median OS: 33.1 months vs. 40.8 months, *p* = 0.0619 and 33.1 months vs. not reached, *p* = 0.0004) (Figure S3A,B). Also, the type of relapse significantly affected patients' outcome, with treatment at symptomatic relapse significantly associated to a worse outcome compared to treatment at biochemical relapse in terms of PFS (13.7 months vs. 23.2 months, *p* = 0.0009) and OS (34.1 months vs. not reached, *p* = 0.0017) (Figure S3C,D).

Considering the type of treatment, anti‐CD38 monoclonal antibody‐based regimen proved to confer improved survival compared to other treatments. In detail PI+anti‐CD38 regimens granted a better PFS compared to IMIDs+anti‐CD38 regimens (23 months vs. 15.4 months, *p* = 0.0805) and especially compared to non‐anti CD38 based regimens (23 months vs. 11.5 months *p* = 0.0039). With respect to OS, no significant difference was observed between PI+anti‐CD38 combinations and IMIDs+anti‐CD38 combinations (not reached vs. 40.8 months, *p* = 0.69) and between PI+anti‐CD38 and non‐anti CD38 based combinations (not reached vs. 39.1 months, *p* = 0.18) (Figure S4A,B).

Finally, we evaluated the PFS and OS of the single regimens used in our cohort. Isa‐KD showed the best PFS results (median PFS 22.8 months), followed by D‐VD (20.12 months), D‐PD (17.29 months), Len‐based triplets (14.5 months), P‐VD (11.77 months) and KD (7.07 months). Considering OS, Isa‐KD and D‐VD confirmed a superior outcome (median OS not reached), followed by Len‐based triplets (44.6 months), D‐PD (40.8 months), P‐VD (33.9 months) and KD (30.7 months) (Figure S4C,D).

Tables S3 and S4 report the results of univariable and multivariable Cox regression analyses on the main variables studied for PFS and OS respectively. For multivariable Cox regression analysis, all significant variables found in univariate analysis were included and a backward elimination was performed to identify factors that retain an independent significant impact on outcome in terms of PFS and OS. HR cytogenetic status, high LDH at relapse and ISS ≥ II at relapse were confirmed to be independently associated with a dismal PFS, while ISS ≥ II at relapse was the best predictor of reduced OS in our cohort of patients.

## DISCUSSION

The data herein presented depict the outcome of a homogeneous cohort of Len‐R MM patients treated at first relapse in the setting of post‐AHSCT maintenance.

In recent years, several randomized trials led to approval of different regimens to be used in the setting of Len‐R patients. However, almost all these trials suffered from large variability of Len‐R patients enrolled, ranging from 24% to 79% of cases and mostly treated at second relapse. In addition, the percentage of patients treated after Len maintenance is not reported.^3^, ^9^, ^11^, ^15^, ^16^, ^17^ In this paper, we described the largest homogenous cohort of patients' refractory to Len maintenance so far reported. The high proportion of patients treated with Isa‐KD reflected both the large proportion of patients who relapsed from 2022 onwards and the assumption that this combination represents the standard of care in this setting, even though the IKEMA trial did not report a median PFS in Len‐R patients. Notably, we report a median PFS of 22.8 months for Isa‐KD in the largest cohort of Len‐R cases treated with Isa‐KD (both in clinical trials and in the real‐life setting). This result is in line with data presented in the CANDOR trial for D‐KD^4^ and with a recent real‐life report.^18^ In our cohort, the D‐PD combination showed a significant median PFS of 17.29 months, which is longer than the 12.4 months reported in the Apollo trial.^11^, ^12^ This can be likely explained considering that in the pivotal trial, the median number of previous treatments was 2–3, while in our study, the patients received only one prior line of therapy (LOT). As a matter of fact, in the phase 2 MM‐014 trial where the median number of LOT was one, the median PFS for D‐PD was even higher (21.8 months).^19^ The prospective phase of the Palladio study is now ongoing aiming to directly compare Isa‐KD and D‐PD regimens, taking into account the different variables (i.e. early relapse, age, high‐risk disease) that can influence the outcome.

Surprisingly, in our cohort D‐VD performed extremely well, with a median PFS of 20.12 months, not so different from Isa‐KD. However, this study has several limitations that should be considered when interpreting the results. The substantial imbalance in sample size between treatment groups, coupled with differences in the timing of patient accrual and follow‐up duration, poses a significant challenge for direct treatment comparisons. Moreover, we cannot exclude that the efficacy of Isa‐KD may be underestimated in this real‐world setting due to a potential enrichment of patients with early, aggressive relapses in that specific subgroup. Certainly, the performance of D‐VD in the context of Len‐R appears particularly favourable in comparison to the data reported in the CASTOR trial. As a matter of fact, several real‐life studies confirmed higher PFS for D‐VD, ranging from 12.9 to 16 months^20^, ^21^, ^22^, ^23^ compared to the 7.8 months reported in the subgroup of Len‐R patients in the pivotal trial.^24^ Among all the treatments, the consistent finding is that anti‐CD38 MoAb‐based regimens (especially PI + anti‐CD38 MoAb combinations) proved to be the best choice compared to anti‐CD38 sparing combinations, with a significant benefit in PFS and an OS advantage.

Unfortunately, the outcome of clinical and biological high‐risk patients is still dismal, for both high tumour burden and genetically high‐risk disease, as unfavourable is the prognosis of patients relapsing early (<12 months) from maintenance initiation. Of note, clinical relapse displays inferior survival, supporting, when possible, starting the treatment at biochemical relapse. However, in recent years the treatment landscape of Len‐R patients has dramatically changed due to the introduction of novel anti‐B cell maturation antigen (BCMA) treatments, providing new therapeutic options for these patients with dismal outcomes. The BCMA‐directed chimeric antigen receptor (CAR)‐T‐cell ciltacabtagene autoleucel (cilta‐cel) proved to be highly effective in Len‐R cases with a median PFS not reached compared to 11.8 months of the standard of care group (mostly D‐PD), with a 12‐month PFS of 75.9%.^25^ Similarly, the combination of the anti‐BCMA antibody‐drug conjugate belantamab mafodotin with pomalidomide and dexamethasone (Bela‐PD) showed superiority to P‐VD in a similar population of relapsed MM patients, with a median PFS of 24 months versus 9.2 months in Len‐R cases.^26^, ^27^ Consequently, the latest European Hematology Association/European Myeloma Network (EMN) MM guidelines recently published recommend cilta‐cel and Bela‐PD at first relapse in Len‐R patients, especially if both anti‐CD38 refractory.^28^ More recently, the combination of the anti‐BCMA bispecific antibody teclistamab with daratumumab proved impressive efficacy in the same setting, with an impressive estimated PFS of 83.4% at 36 months.^29^

In conclusion, we presented the outcome of a homogenous cohort of Len‐R patients treated at first relapse after maintenance. Anti‐CD38 MoAb‐based combinations proved to be the standard of care regimens in this setting, in particular the combinations of PI+anti‐CD38, with Isa‐KD showing the longest PFS. However, high‐risk disease still represents an unmet clinical need, with dismal outcome. In this setting, the novel anti‐BCMA treatments, including CAR‐T and bispecific antibodies, will provide the new standard of care for the treatment of this difficult to treat population.

## AUTHOR CONTRIBUTIONS

**Angela Grassi:** Writing – original draft; methodology; formal analysis; data curation. **Edoardo Olivari:** Data curation. **Claudio Salvatore Cartia:** Data curation. **Francesca Fazio:** Data curation; writing – review and editing. **Sonia Morè:** Data curation; writing – review and editing. **Carmine Liberatore:** Data curation; writing – review and editing. **Martina Tinelli:** Data curation. **Concetta Conticello:** Data curation; writing – review and editing. **Emanuele Favero:** Data curation. **Giuseppe Mele:** Data curation. **Valeria Ruocco:** Data curation. **Martina Gherardini:** Data curation. **Massimo Gentile:** Data curation. **Enrica Antonia Martino:** Data curation. **Nicola Sgherza:** Data curation. **Edoardo Scomazzon:** Data curation. **Roberto Mina:** Data curation; writing – review and editing. **Marco Talarico:** Data curation; writing – review and editing. **Bernardo Rossini:** Data curation. **Ombretta Annibali:** Data curation. **Gregorio Barilà:** Conceptualization; investigation; writing – original draft; data curation; formal analysis. **Laura Paris:** Data curation. **Claudio De Magistris:** Data curation. **Francesca Fioritoni:** Data curation. **Maria Livia Del Giudice:** Data curation. **Antonia Cagnetta:** Data curation. **Valeria Tomarchio:** Data curation. **Maria Luisa Pioltelli:** Data curation. **Giuseppe Pietrantuono:** Data curation. **Massimiliano Arangio Febbo:** Data curation. **Anna Furlan:** Data curation; writing – review and editing. **Anna Maria Cafro:** Data curation. **Paola Boggione:** Data curation. **Francesco Vassallo:** Data curation. **Sara Pezzatti:** Data curation. **Maria Teresa Petrucci:** Writing – review and editing; conceptualization; supervision. **Francesco Pisani:** Data curation. **Elena Zamagni:** Writing – review and editing. **Monica Galli:** Data curation; writing – review and editing. **Pellegrino Musto:** Data curation; writing – review and editing. **Roberta Della Pepa:** Data curation. **Lorenzo De Paoli:** Data curation. **Silvia Mangiacavalli:** Data curation; writing – review and editing. **Elisabetta Antonioli:** Data curation. **Alberto Tosetto:** Writing – review and editing; data curation. **Massimo Offidani:** Supervision; writing – review and editing; conceptualization. **Gabriele Buda:** Data curation. **Renato Zambello:** Writing – review and editing; conceptualization; supervision. **Francesca Patriarca:** Writing – review and editing; data curation.

## FUNDING INFORMATION

No funding was received for conducting this study.

## CONFLICT OF INTEREST STATEMENT

GB has received honoraria from Amgen, Menarini‐Stemline, Sanofi, Bristol‐Myers Squibb and Johnson & Johnson, has served on the advisory boards for Johnson & Johnson, Sanofi, Pfizer and Menarini‐Stemline and has received consultancy fees from Johnson & Johnson, Sanofi and Pfizer. FP has served on the advisory boards and honoraria from Glaxo‐Smith‐Kline, Amgen, Johnson &Johnson, Menarini‐Stemline, Pfizer. PM has received honoraria for lectures and/or participation to advisory board memberships, including travels and hotel accommodations, with: Abbvie, Alexion, Amgen, Astellas, Astra‐Zeneca, Bei‐Gene, Bristol‐Myers Squibb, Daichi‐Sankyo, Eli‐Lilly, Gilead, Glaxo‐Smith‐Kline, Grifols, Incyte, Johnson & Johnson, Jazz, Kyowa Kirin, Menarini‐Stemline, Otsuka, Novartis, Pfizer, Roche, Sanofi, Servier, Sobi and Takeda. RZ has served on advisory board for Amgen, Takeda, Bristol‐Myers Squibb, Johnson &Johnson, Sanofi and Pfizer. All the other authors have no relevant financial or non‐financial interests to disclose.

## ETHICS STATEMENT

All procedures performed in studies involving human participants were in accordance with the ethical standards of the institutional and/or national research committee and with the 1964 Helsinki Declaration and its later amendments or comparable ethical standards. The study was approved by Local Ethical Committee of the South‐West Veneto (CET‐ASOV, protocol number VI‐MM‐02).

## INFORMED CONSENT

Informed consent was obtained from all individual participants included in the study.

## Supporting information


**Table S1.** Table corresponding to the stacked barplot in Figure 1.
**Table S2.** Response rates for each therapy combination at relapse.
**Table S3.** Univariable and multivariable Cox proportional hazards regression analyses for PFS.
**Table S4.** Univariable and multivariable Cox proportional hazards regression analyses for OS.
**Figure S1.** Progression‐free survival and overall survival by cytogenetic risk.
**Figure S2.** Progression‐free survival and overall survival in patients with reduce renal function and extra‐medullary disease.
**Figure S3.** Progression‐free survival and overall survival with respect to the relapse.
**Figure S4.** Progression‐free survival and overall survival by type of treatment.

## Data Availability

The data that support the findings of this study are available on request from the corresponding author. The data are not publicly available due to privacy or ethical restrictions.
