# Supplementary material for: Salvage treatment patterns in multiple myeloma patients progressing after lenalidomide maintenance: A real‐life study from European Myeloma Network (EMN) Italy
Source: Br J Haematol. 2026 Aug 18;209(4):1704–14. doi: 10.1111/bjh.70751 (PMC13674119; doi:10.1111/bjh.70751)
Supplement: Supplementary file 1 [file BJH-209-1704-s001.docx]

**SUPPLEMENTARY**

**Supplementary Table 1: Table corresponding to the stacked barplot in Figure 1.**

| **Year** | **N** | **Isa-KD** | **D-PD** | **D-VD** | **P-VD** | **KD** | **Len-based triplets** | **Others** |
| --- | --- | --- | --- | --- | --- | --- | --- | --- |
| 2017 | 1 |  |  |  |  |  | 1 (100%) |  |
| 2018 | 3 |  |  | 2 (66.7%) |  |  |  | 1 (33.3%) |
| 2019 | 14 |  | 1 (7.1%) | 9 (64.3%) |  | 3 (21.4%) |  | 1 (7.1%) |
| 2020 | 18 |  | 2 (11.1%) | 8 (44.4%) |  | 4 (22.2%) | 3 (16.7%) | 1 (5.6%) |
| 2021 | 47 |  | 4 (8.5%) | 14 (29.8%) | 16 (34%) | 9 (19.1%) | 4 (8.5%) |  |
| 2022 | 73 | 51 (69.9%) | 1 (1.4%) | 4 (5.5%) | 7 (9.6%) | 2 (2.7%) | 8 (11.0%) |  |
| 2023 | 81 | 45 (55.6%) | 32 (39.5%) |  |  | 1 (1.2%) | 2 (2.5%) | 1 (1.2%) |
| 2024 | 67 | 42 (62.7%) | 19 (28.4%) | 1 (1.5%) | 1 (1.5%) |  | 2 (3.0%) | 2 (3.0%) |
| 2025 | 1 | 1 (100%) |  |  |  |  |  |  |

N: number of patients.

**Supplementary Table 2: Response rates for each therapy combination at relapse.**

| **Therapy** | **N** | **sCR** | **CR** | **VGPR** | **PR** | **MR** | **SD** | **PD** |
| --- | --- | --- | --- | --- | --- | --- | --- | --- |
| **Isa-KD** | 134 | 14 (10.4%) | 28 (20.9%) | 39 (29.1%) | 33 (24.6%) |  | 14 (10.4%) | 6 (4.5%) |
| **D-PD** | 57 | 3 (5.3%) | 12 (21.1%) | 13 (22.8%) | 22 (38.6%) | 1 (1.8%) | 4 (7.0%) | 2 (3.5%) |
| **D-VD** | 38 |  | 10 (26.3%) | 13 (34.2%) | 3 (7.9%) |  | 8 (21.1%) | 4 (10.5%) |
| **P-VD** | 24 | 1 (4.2%) | 3 (12.5%) | 7 (29.2%) | 10 (41.7%) |  | 2 (8.3%) | 1 (4.2%) |
| **KD** | 19 | 1 (5.3%) | 2 (10.5%) | 4 (21.1%) | 6 (31.6%) | 2 (10.5%) | 3 (15.8%) | 1 (5.3%) |
| **Len-based triplets** | 20 | 0 | 6 (30.0%) | 5 (25.0%) | 7 (35.0%) | 1 (5.0%) | 1 (5.0%) | 0 |
| **Others** | 6 | 0 | 1 (16.7%) | 2 (33.3%) | 1 (16.7%) | 1 (16.7%) | 1 (16.7%) | 0 |

N: number of patients.

**Supplementary Table 3: Univariable and multivariable Cox proportional hazards regression analysis for PFS.**

|  | **Univariable analysis PFS** | | | **Multivariable analysis PFS** | | |
| --- | --- | --- | --- | --- | --- | --- |
| **Characteristic** | **HR**^1^ | **95% CI**^1^ | **p-value** | **HR**^1^ | **95% CI**^1^ | **p-value** |
| **Gender Male** | 1.00 | 0.73, 1.37 | >0.9 |  |  |  |
| **Age at treatment initiation after relapse >65 years** | 0.82 | 0.59, 1.13 | 0.2 |  |  |  |
| **Anti-CD38 based treatment** | 0.80 | 0.30, 2.18 | 0.7 |  |  |  |
| **Relapse time from maintenance initiation >24 months** | 0.66 | 0.48, 0.93 | **0.016** |  |  |  |
| **HR Cytogenetic status at relapse [del(17p),t(4;14),t(14;16),+1q]** | 1.87 | 1.08, 3.23 | **0.025** | 2.69 | 1.15, 6.32 | **0.023** |
| **Cytogenetic abnormalities ≥2** | 1.58 | 0.94, 2.67 | 0.085 |  |  |  |
| **High LDH at relapse** | 1.98 | 1.36, 2.87 | **<0.001** | 2.66 | 1.22, 5.83 | **0.014** |
| **ISS II-III at relapse** | 2.81 | 1.82, 4.34 | **<0.001** | 2.42 | 1.19, 4.94 | **0.015** |
| **Symptomatic relapse** | 1.78 | 1.26, 2.51 | **<0.001** |  |  |  |
| **eGFR≥60** | 0.70 | 0.47, 1.06 | 0.092 |  |  |  |
| **EMD at relapse** | 1.56 | 0.84, 2.89 | 0.2 |  |  |  |

*^1^*HR = Hazard Ratio, CI = Confidence Interval.

*Abbreviations: HR: High Risk. eGFR: estimated glomerular filtration rate. ISS: international staging system. EMD: extramedullary disease.*

**Supplementary Table 4: Univariable and multivariable Cox proportional hazards regression analysis for OS.**

|  | **Univariable analysis OS** | | | **Multivariable analysis OS** | | |
| --- | --- | --- | --- | --- | --- | --- |
| **Characteristic** | **HR**^1^ | **95% CI**^1^ | **p-value** | **HR**^1^ | **95% CI**^1^ | **p-value** |
| **Gender Male** | 1.12 | 0.71, 1.75 | 0.6 |  |  |  |
| **Age at treatment initiation after relapse >65 years** | 1.07 | 0.68, 1.68 | 0.8 |  |  |  |
| **Anti-CD38 based treatment** | 0.98 | 0.24, 4.00 | >0.9 |  |  |  |
| **Relapse time from maintenance initiation >24 months** | 0.44 | 0.25, 0.76 | **0.003** | 0.526 | 0.25, 1.11 | 0.093 |
| **Cytogenetic status at relapse HR [del(17p),t(4;14),t(14;16),+1q]** | 1.00 | 0.48, 2.08 | >0.9 |  |  |  |
| **Cytogenetic abnormalities ≥2** | 1.33 | 0.61, 2.89 | 0.5 |  |  |  |
| **LDH at relapse High** | 2.89 | 1.79, 4.66 | **<0.001** |  |  |  |
| **ISS II-III at relapse** | 4.66 | 2.45, 8.87 | **<0.001** | 4.732 | 2.49, 9.00 | **<0.001** |
| **Type of relapse Symptomatic** | 2.32 | 1.35, 3.97 | **0.002** |  |  |  |
| **eGFR≥60** | 0.49 | 0.29, 0.81 | **0.006** |  |  |  |
| **EMD at relapse** | 0.97 | 0.35, 2.67 | >0.9 |  |  |  |

*^1^*HR = Hazard Ratio, CI = Confidence Interval.

*Abbreviations: HR: High Risk. eGFR: estimated glomerular filtration rate. ISS: international staging system. EMD: extramedullary disease.*

**Supplementary Figure 1: Progression Free Survival and Overall Survival by cytogenetic risk.
Panel A** Progression Free Survival (PFS) and **Panel B** Overall Survival (OS) according to cytogenetic risk stratification at relapse, into standard risk (SR), isolated +1q and high risk (HR) including del(17p), t(4;14) and t(14;16). Survival curves were estimated using the Kaplan–Meier method and compared by log-rank test.

**A**

**B**

**Supplementary Figure 2: Progression Free Survival and Overall survival in patients with reduce renal function and extramedullary disease.**

**Panels A and B:** PFS (Panel A) and OS (Panel B) depending on eGFR. **Panels C and D:** PFS (Panel C) and OS (Panel D) depending on the presence of extramedullary disease (EMD). Survival curves were estimated using the Kaplan–Meier method and compared by log-rank test.

**Supplementary Figure 3:** **Progression Free Survival and Overall survival with respect to the relapse.**

**Panels A and B:** PFS (Panel A) and OS (Panel B) according to the duration (in months) of previous lenalidomide maintenance. **Panels C and D:** PFS (Panel C) and OS (Panel D) according to the type of relapse. Survival curves were estimated using the Kaplan–Meier method and compared by log-rank test.

**Supplementary Figure 4:** **Progression Free Survival and Overall Survival by type of treatment.**

**Panels A and B:** PFS (Panel A) and OS (Panel B) depending on the second line treatment combination used in the study cohort. **Panels C and D:** PFS (Panel C) and OS (Panel D) according to the different second line regimens used in the study cohort. Survival curves were estimated using the Kaplan–Meier method and compared by log-rank test.
